# Global incidence and case fatality rate of pulmonary embolism following major surgery: a protocol for a systematic review and meta-analysis of cohort studies

**DOI:** 10.1186/s13643-017-0647-8

**Published:** 2017-12-04

**Authors:** Mazou N. Temgoua, Joel Noutakdie Tochie, Jean Jacques Noubiap, Valirie Ndip Agbor, Celestin Danwang, Francky Teddy A. Endomba, Njinkeng J. Nkemngu

**Affiliations:** 1 Department of Medicine and sub-Specialties, Faculty of Medicine and Biomedical Sciences, University of Yaounde I, Yaounde, Cameroon; 2 Department of Surgery and sub-Specialties, Faculty of Medicine and Biomedical Sciences, University of Yaounde I, Yaounde, Cameroon; 3 Department of Medicine, Groote Schuur Hospital and University of Cape Town, 7925 Observatory, Cape Town, South Africa; 4 Ibal sub-Divisional Hospital, Oku, North-west Region Cameroon; 5 Department of Anesthesia, University of Toronto, Toronto, Canada

**Keywords:** Pulmonary embolism, Major surgery, Incidence, Mortality, Determinants, Cohort studies

## Abstract

**Background:**

Pulmonary embolism (PE) is a life-threatening condition common after major surgery. Although the high incidence (0.3–30%) and mortality rate (16.9–31%) of PE in patients undergoing major surgical procedures is apparent from findings of contemporary observational studies, there is a lack of a summary and meta-analysis data on the epidemiology of postoperative PE in this same regard. Hence, we propose to conduct the first systematic review to summarise existing data on the global incidence, determinants and case fatality rate of PE following major surgery.

**Methods:**

Electronic databases including MEDLINE, EMBASE, SCOPUS, WHO global health library (including LILACS), Web of Science and Google scholar from inception to April 30, 2017, will be searched for cohort studies reporting on the incidence, determinants and case fatality rate of PE occurring after major surgery. Data from grey literature will also be assessed. Two investigators will independently perform study selection and data extraction. Included studies will be evaluated for risk of bias. Appropriate meta-analytic methods will be used to pool incidence and case fatality rate estimates from studies with identical features, globally and by subgroups of major surgical procedures. Random-effects and risk ratio with 95% confidence interval will be used to summarise determinants and predictors of mortality of PE in patients undergoing major surgery.

**Discussion:**

This systematic review and meta-analysis will provide the most up-to-date epidemiology of PE in patients undergoing major surgery to inform health authorities and identify further research topics based on the remaining knowledge gaps.

**Systematic review registration:**

PROSPERO CRD42017065126

**Electronic supplementary material:**

The online version of this article (10.1186/s13643-017-0647-8) contains supplementary material, which is available to authorized users.

## Background

Globally, about 234 million major surgical interventions are performed each year [1] and postoperative complications inflict a significant morbidity, mortality and economic burden to surgical patients as well as healthcare systems [2, 3]. Pulmonary embolism (PE), a life-threatening complication of venous thromboembolism (VTE), represents a major postoperative complication [4]. Indeed, surgery increases the risk of PE by fivefold [5]. The pathogenesis involves the interplay of an acute inflammatory reaction triggered by vascular endothelial lesions due to surgical dissections [6], hypercoagulability induced by surgical stress and anaesthetic drugs [7, 8], and venous stasis due to perioperative immobilisation and delayed rehabilitation, all described by Virchow more than a century ago [9]. PE is known to be relatively frequent during major surgery, where the following incidence rates have been reported: 0.7–30% after orthopaedic surgery [10], 1.5–7.6% following thoracic surgery [11, 12], 0.33–6.6% after abdominal surgery [13, 14], 0.3–4.1% in gynaecologic surgery [15–17], 0.9–1.1% after urologic procedures [18, 19], and 0.6% after cardiac surgery [20]. Furthermore, despite the widespread adoption of thromboembolic prophylactic measures, PE accounts for a 30-day major surgery-specific case fatality rate ranging between 16.9 and 31% [13, 21–23] and a 37% [21] 1-year case fatality rate following major surgery.

In spite of recent advances in the diagnosis of PE, notably with the advent of multi-detector computed tomography pulmonary angiography and ventilation-perfusion scintigraphy [4], PE remains the most common cause of preventable death in surgical patients that is often easily overlooked [24–26]. Furthermore, these diagnostic imaging tests [4] have been described to be inaccessible for the majority of patients with suspected PE in resource-limited settings [27]. While several patients with PE remain asymptomatic, others will experience a fatal PE as the first manifestation of thromboembolism [28]. Therefore, identifying perioperative risk factors of PE is an important step towards prevention of PE in patients undergoing major surgery, primarily via prompt thromboembolic prophylaxis [29, 30]. Hence, this protocol is designed for a systematic review and meta-analysis to critically synthesise the global incidence, determinants and case fatality rate of PE following major surgery. Findings will provide evidence on the current epidemiology of PE following major surgery and inform policymakers on major determinants of PE for which control interventions can be tailored to curb this burden.

## Objectives

The aim of this systematic review and meta-analysis is to ascertain the global incidence, determinants and case fatality rate of PE after major surgery.

## Review questions

To this end, the proposed systematic review will answer the following questions:

1. What is the global incidence of PE in patients undergoing major surgery?
2. What are the determinants of PE among patients undergoing major surgery?
3. What is the specific case fatality rate for PE after major surgery?

## Methods and design

This review is reported in accordance with the Preferred Reporting Items for Systematic Review and Meta-Analysis Protocols (PRISMA-P) 2015 Guidelines [31] and applicable to observational studies. An additional file shows this in more detail (see Additional file 1).

### Criteria for considering studies for the review

#### Inclusion criteria

1. We intend to include cohort studies reporting any of the following three data: incidence, determinants, and case fatality rate of PE following major surgical procedures round the world. Cohort studies that report other postoperative complications including PE will be included provided they present data enabling the calculation of any of the aforementioned three endpoints.
2. Study participants will be all patients who underwent a major surgical intervention. Major surgery will be defined as any intervention involving the incision, resection, manipulation or suturing of tissue and that requires general or regional anaesthesia or profound sedation for analgesia [32].
3. The diagnosis of PE will be based on computed tomographic pulmonary angiography and ventilation-perfusion scintigraphy as defined by the European Society of Cardiology [33] and the American Heart Association [34]. Studies reporting PE based on histopathology diagnoses made at autopsy shall also be considered. We intend to consider all published and unpublished data from inception to April 30, 2017.
4. Study settings: health care facilities in either rural or urban settings.
5. No language restriction.

#### Exclusion criteria

1. Studies where there are no appropriate diagnostic tool for PE.
2. For duplicate publications, only the publication with the most person-years will be retained.

### Search strategy for study identification

We intend to search MEDLINE, EMBASE, SCOPUS, WHO global health library (including LILACS), Web of Science and Google scholar from January 1, 1997, to April 30, 2017, for published studies on PE in adults undergoing major surgery. A comprehensive search strategy combining key search terms of different major surgical procedures cross-referenced with pulmonary embolism will be used in order to obtain the maximum possible number of studies. The search strategy for MEDLINE/PubMed is shown in Table 1. This will be adapted for the other databases. We will scan the reference lists of retrieved articles in order to identify any additional relevant studies. Our search will extend to include grey literature from book chapters, conference proceedings, theses, non-governmental organisations and government reports.

**Table 1:** Search strategy for MEDLINE and adaptability to all databases

Search term	Search terms	Hits
1	(“Non-cardiac surgery” [tw] OR “Non-cardiac surgical procedure” [tw] OR “Gynaecologic surgery” [tw] OR “Gynecologic surgery” [tw] OR “Hysterectomy” [tw] OR “Caesarean section” [tw] OR “Caesarean delivery” [tw] OR “Wertheim” [tw] OR “Ovarian cystectomy [tw] OR “Myomectomy [tw] OR “Fimbrioplasty [tw] OR “Cervicectomy” [tw] OR “Oophorectomy” [tw] OR “Salpingoophrorectomy” [tw] OR “Salpingectomy” [tw] OR “Vaginectomy” [tw] OR “Vulvectomy” [tw] OR “Salpingostomy” [tw] OR “Hysterotomy” [tw] OR “Abdominal Surgery” [tw] OR “Visceral surgery” [tw] OR “Laparotomy” [tw] OR “Oesophagectomy” [tw] OR “Nissen fundoplication” [tw] OR “Hernia repair” [tw] OR “Herniorraphy” [tw] OR “Appendectomy” [tw] OR “Appendicectomy” [tw] OR “cholecystectomy” [tw] OR “Colectomy” [tw] OR “Colostomy” [tw] OR “Gastrectomy” [tw] OR “Gastrotomy” [tw] OR “Gastroplasty” [tw] OR “Gastroduodenostomy” [tw] OR “Gastroenterostomy” [tw] OR “Ileostomy” [tw] OR “Jejunostomy” [tw] OR “Colostomy” [tw] OR “Cholecystostomy” [tw] OR “Splenectomy” [tw] OR “Splenoplexy” [tw] OR “Whipple” [tw] OR “Pancreatectomy” [tw] OR “Pancreatoduodenectomy” [tw] OR “Protocolectomy” [tw] OR “Omentopexy” [tw] OR “Hepatectomy” [tw] OR “Hepatoportoenterostomy” [tw] OR “Sigmoidostomy” [tw] OR “Sphinterotomy” [tw] OR “Pyloromyotomy” [tw] OR “Hemorrhoidectomy” [tw] OR “Urology surgery” [tw] OR “Nephrectomy” [tw] OR “Lithotomy” [tw] OR “Urinary Cystectomy” [tw] OR “Nephrostomy” [tw] OR “Ureterostomy” [tw] OR “Nephrotomy” [tw] OR “Nephropexy” [tw] OR “Urethropexy” [tw] OR “Kidney transplantation” [tw] OR “Adenectomy” [tw] OR “Prostatectomy” [tw] OR “Urethroplasty” [tw] OR “Pyeloplasty” [tw] OR “Orthopedic surgery” [tw] OR “Osteosynthesis” [tw] OR “Fracture fixation” [tw] OR “Fracture stabilisation” [tw] OR “Internal fixation” [tw] OR “External fixation” [tw] OR “Hip replacement” [tw] OR “Knee replacement” [tw] OR “Arthroplasty” [tw] OR “Ostectomy” [tw] OR “Osteotomy” [tw] OR “Arthrotomy” [tw] OR “Laminotomy” [tw] OR “Foraminotomy” [tw] OR “Epiphysiodesis” [tw] OR “Arhrodesis” [tw] OR “Ligament reconstruction” [tw] OR “Limb Amputation” [tw] OR “Fasciotomy” [tw] OR “Prosthestic surgery” [tw] OR “Prosthesis” [tw] OR “Spinal Surgery” [tw] OR “Back Surgery” [tw] OR “Facetectomy” [tw] OR “Laminectomy” [tw] OR “Discectomy” [tw] OR “Thoracotomy” [tw] OR “Thoracostomy” [tw] OR “Pneumectomy” [tw] OR “Pneomotomy” [tw] OR “Lobectomy” [tw] OR “Bronchotomy” [tw] OR “Tracheotomy” [tw] OR “Pleuridesis” [tw] OR “Lung transplantation” [tw] OR “Neurosurgery” [tw] OR “Craniotomy” [tw] OR “Craniectomy” [tw] OR “Hemispherectomy” [tw] OR “Ureterosigmoidostomy” [tw] OR “Fistulotomy” [tw] OR “Cerebral lobectomy” [tw] OR “Cerebral lobotomy” [tw] OR “Hypophysectomy” [tw] OR “Amydalohippocampectomy” [tw] OR “Ventriculostomy” [tw] OR “Ventriculoperitoneal shunting” [tw] OR “Pallidotomy” [tw] OR “Thalamotomy” [tw] OR “Cingulotomy” [tw] OR “Cordotomy” [tw] OR “Rhizotomy” [tw] OR “Brain biopsy” [tw] OR “Ganglionectomy” [tw] OR “Symapathectomy” [tw] OR “Neurectomy” [tw] OR “Axotomy” [tw] OR “Vagotomy” [tw] OR “Nerve biopsy” [tw] OR “Endocrine surgery” [tw] OR “Thyroidectomy” [tw] OR “Thyroid lobectomy” [tw] OR “Parathyroidectomy” [tw] OR “Adrenalectomy” [tw] OR “Pinealectomy” [tw] OR “Ophthalmic surgery” [tw] OR “Punctoplasty” [tw] OR “Trabeculopathy” [tw] OR “Photorefractive keratectomy” [tw] OR “Trabeculectomy” [tw] OR “Iridectomy” [tw] OR “Virectomy” [tw] OR “Dacryocystorhinostomy” [tw] OR “Radial keratotomy” [tw] OR “Corneal transplantation” [tw] OR “ENT surgery” [tw] OR “Ear nose throat surgery” [tw] OR “Otoplasty” [tw] OR “Stapedectomy” [tw] OR “Mastoidectomy” [tw] OR “Auriculectomy” [tw] OR “Myringotomy” [tw] OR “Rhinoplasty” [tw] OR “Septoplasty” [tw] OR “Rhinectomy” [tw] OR “Laryngectomy” [tw] OR “Tracheostomy” [tw] OR “Sinusotomy” [tw] OR “Cricothyroidotomy” [tw] OR “Cricothyrotomy Tonsillectomy” [tw] OR “Adenoidectomy” [tw] OR “Palatoplasty” [tw] OR “Uvuloplasty” [tw] OR “Gingivectomy” [tw] OR “Glossectomy” [tw] OR “Cardiac surgery” [tw] OR “Valvuloplasty” [tw] OR “Pericardiectomy” [tw] OR “Pericardiotomy” [tw] OR “Cardiotomy” [tw] OR “Coronary artery bypass surgery” [tw] OR “Heart transplantation” [tw] OR “Vascular surgery” [tw] OR “Angioplasty” [tw] OR “Endarectomy” [tw] OR “Lympahatic surgery” [tw] OR “Thymectomy” [tw] OR “Lymphadenectomy” [tw] OR “Thymus transplantation” [tw] OR “Spleen transplantation” [tw])	
2	Pulmonary embolism [tw] OR Pulmonary emboli [tw] OR Thromboembolism [tw] OR Thromboembolic events [tw] OR Thromboembolic complications [tw] OR Venous thromboembolism [tw]	
3	#1 AND #2 Limits: 01/01/1997 to 31/04/2017	

BTW70647BTW70647

### Study records

#### Data management

All studies identified by the search strategy will be entered into EndNote to assess and exclude duplication of records. This will be subsequently uploaded into Eppi-Reviewer, an Internet-based software program which eases collaboration among reviewer authors during the study selection process. Prior to the screening of studies, the team will develop and screen questions and forms for two levels of assessment based on the eligibility criteria. Abstracts and full-text articles will be uploaded with screening questions to Eppi-Reviewer.

#### Study screening and selection

Two review authors (JNT and MNT) will independently screen the titles and abstracts of studies yielded by the search against the eligibility criteria. We shall retrieve full reports for all study titles that appear to meet the eligibility criteria or where there is any contingency. The investigators will then screen the full-text reports and decide whether these meet the inclusion criteria of the systematic review. We intend to seek additional data from corresponding authors in the event that the publication is unclear and hence may be subject to multiple interpretations. Discrepancies will be solved by a third author (NJN). We will record the reasons for excluding studies.

#### Data extraction

With the aid of a standardised and pre-tested data extraction form, two investigators (VNA and CD) will extract data independently from included studies. Any disagreement shall be resolved by discussion, and a third investigator (FTAE) will adjudicate unresolved disagreements. A flowchart will be used to demonstrate the entire study selection and data extraction processes.

#### Data items

We will seek information concerning the following study variables: the year the study was conducted and published, the language of publication, demographic data of participants (mean age, gender proportions), setting (urban or rural hospital), sample size, number and proportion with PE, type of major surgical procedure, risk factors for PE, PE-related mortality rate, predictors for PE-related mortality and measures of association (relative risks, odds ratios, *p* values and confidence intervals) will be recorded. If measures of association cannot be calculated or are not reported in the primary study, we shall contact the concerned authors via email for additional information.

#### Assessment of risk of bias

To assess the risk of bias within included studies, two review authors (JJN and MNT) will independently assess the risk of bias within included studies using the Newcastle-Ottawa Scale (NOS) [35]. An additional file shows this scale in detail (see Additional file 2). A judgement as to the possible risk of bias in each study will be rated as ‘high risk’, ‘moderate risk’ or ‘low risk’. In the event of insufficient details reported in a study, we will score the risk of bias as ‘uncertain’ and the investigators of the said primary study will be contacted for more information. We intend to present the risk of bias in a table. Also, we will compute graphic representations of potential bias within and across studies using RevMan V.5.3. Disagreements will be resolved first by discussion and then by consulting a third author (NJN) for arbitration.

#### Data synthesis, analysis and assessment of heterogeneity

Data on the incidence, case fatality rate and determinants of PE following major surgery will be summarised in demographic characteristic (patient age, sex), clinical characteristics (type of major surgery, pregnancy, obesity, prior venous thromboembolism, active malignancy, infection, intensive care unit admission, trauma or fracture, hormonal therapy, immobilisation), study setting and the time the study was conducted and published. Heterogeneity will be assessed using the chi-square (*χ* ^2^) test on Cochran’s *Q* statistic and quantified by calculating the *I*-squared (*I*_2_) statistic [36]. *I*_2_ values of 25, 50 and 75% will represent low, medium and high heterogeneity, respectively. In the event of no substantial heterogeneity across studies, a meta-analysis will be performed for the global incidence and case fatality rates of PE following major surgery across studies with similar characteristics. To this end, a random effects meta-analysis model will be used to obtain an overall summary estimate of the incidence and mortality rate across included studies. Furthermore, an inverse variance weighting method will be used for a meta-analysis of risk ratios. Where substantial heterogeneity will be detected despite lumping similar studies, subgroup analyses will be conducted to explore the possible sources of heterogeneity using the following grouping parameters: gender, age, study setting, the time the study was conducted and published, diagnostic methods and type of major surgery. In the event that the data cannot be pooled due to substantial heterogeneity, only a narrative summary of the findings will be done.

#### Assessment of reporting bias

We will assess publication bias and selective reporting by using funnel plots and Egger’s test. Data will be analysed using Stata software version 13 (Stata Corp V.13, TX, USA).

#### Protocol amendments

In order to limit outcome reporting bias, we do not intend to make any amendment to this protocol. However, in the event of any amendment, we will report the date of the amendment, a description of the modification done and the rationale. Only protocol amendments approved by all the authors will be documented by the guarantor.

## Discussion

Pulmonary embolism is a life-threatening complication of deep venous thrombosis. The risk of incidence is increased by major surgery. A summary and meta-analysis of contemporary cohort studies reporting its incidence, determinants and case fatality rate in patients undergoing major surgery will be invaluable in assessing its true impact in the perioperative period, and informing health authorities on major determinants of PE for which control interventions can be geared to curb this burden.

## Additional files

Additional file 1: Preferred Reporting Items for Systematic Review and Meta-Analysis Protocols (PRISMA-P) 2015 Guidelines (DOCX 17 kb)

Additional file 2: Newcastle-Ottawa Scale (DOCX 14 kb)

## Abbreviations

DVT: Deep vein thrombosis
PE: Pulmonary embolism
VTE: Venous thromboembolism
