# Supplementary material for: Global incidence and case fatality rate of pulmonary embolism following major surgery: a protocol for a systematic review and meta-analysis of cohort studies
Source: Syst Rev. 2017 Dec 4;6:240. doi: 10.1186/s13643-017-0647-8 (PMC5716368; doi:10.1186/s13643-017-0647-8)
Supplement: Supplementary file 2 — Newcastle-Ottawa Scale (DOCX 14 kb) [file 13643_2017_647_MOESM2_ESM.docx]

NEWCASTLE - OTTAWA QUALITY ASSESSMENT SCALE
COHORT STUDIES

Note: A study can be awarded a maximum of one star for each numbered item within the Selection and Outcome categories. A maximum of two stars can be given for Comparability.

Selection
1) Representativeness of the exposed cohort
a) Truly representative of the average _______________ (describe) in the community ⃰
b) Somewhat representative of the average ______________ in the community ⃰
c) Selected group of users eg nurses, volunteers
d) No description of the derivation of the cohort
2) Selection of the non-exposed cohort
a) Drawn from the same community as the exposed cohort ⃰
b) Drawn from a different source
c) No description of the derivation of the non-exposed cohort
3) Ascertainment of exposure
a) Secure record (eg surgical records) ⃰
b) Structured interview ⃰
c) Written self-report
d) No description
4) Demonstration that outcome of interest was not present at start of study
a) Yes ⃰
b) No
Comparability
1) Comparability of cohorts on the basis of the design or analysis
a) Study controls for _____________ (select the most important factor) ⃰
b) Study controls for any additional factor ⃰ (This criteria could be modified to indicate specific
control for a second important factor.)
Outcome
1) Assessment of outcome
a) Independent blind assessment ⃰
b) Record linkage ⃰
c) Self-report
d) No description
2) Was follow-up long enough for outcomes to occur
a) Yes (select an adequate follow up period for outcome of interest) ⃰
b) No
3) Adequacy of follow up of cohorts
a) Complete follow up - all subjects accounted for ⃰
b) Subjects lost to follow up unlikely to introduce bias - small number lost - > ____ % (select an
adequate %) follow up, or description provided of those lost) ⃰
c) Follow up rate < ____% (select an adequate %) and no description of those lost
d) No statement
